# That Two Hundred Dollar Prize

**Published:** 1887-09

**Authors:** 


### THAT TWO HUNDRED DOLLAR PRIZE.

Every honest man will, at heart, probably, sincerely rejoice that the American Dental Association, at its late meeting, removed what many members believed was a dark stain upon its escutcheon. In 1882, on the recommendation of the President, Dr. H. A. Smith, the Society offered a prize of two hundred dollars, "to be awarded by a special committee of three members of this Association, to be appointed by the President, to the author of the best paper upon the Etiology of Dental Caries, the paper to be based upon strictly original investigation, and to be presented to this Association in the report of Section VII." (Note the language used.)

At the next annual meeting, the committee, consisting of Drs. C. N. Pierce, E. T. Darby and H. A. Smith, presented a paper by Dr. W. D. Miller, of Berlin, and closed their report as follows: "Your committee would, therefore, in view of the original work which the author has prosecuted during the past two years, the results of which are given in this paper, award (*sic*) to the essayist the two hundred dollars appropriated for this purpose."

The report, at a very full session, was unanimously adopted. At the last session of the same meeting, when nearly all the members had gone home, through some fatuity or misunderstanding the vote was, by the few then present, reconsidered and reversed. This, by the terms of the original resolution and the definite and specific report of the committee to whom was delegated the power to make the award, the Society had no right to do, but to avoid controversy the vote was not questioned.

At the late meeting, the same Section, in its report, reviewed the whole matter, and recommended that the original award be complied with, and the money paid in accordance with the report of its special committee, and this recommendation was unanimously adopted. Tardy justice has thus been done, and the honor of the Society redeemed.

